# Supplementary material for: MGDrivE 3: A decoupled vector-human framework for epidemiological simulation of mosquito genetic control tools and their surveillance
Source: PLoS Comput Biol. 2024 May 28;20(5):e1012133. doi: 10.1371/journal.pcbi.1012133 (PMC11161092; doi:10.1371/journal.pcbi.1012133)
Supplement: S1 Table — (PDF) [file pcbi.1012133.s001.pdf]

Table S1. Model parameters describing the gene drive construct, mosquito bionomics and malaria epidemiology for simulations resembling releases on São Tomé, São Tomé and Príncipe.

Symbol:	Parameter:	Value:	Reference:
**Gene drive construct:**			
f_{HH}	Homozygous fitness (relative to wildtype) on female mosquitoes	0.9	[1]
f_H	Hemizygous fitness (relative to wildtype) on female mosquitoes	0.9	[1]
m_{HH}	Homozygous male mating competitiveness	1.05	[1]
m_H	Hemizygous male mating competitiveness	1.78	[1]
$p^M{}_W$	Probability of wildtype allele staying intact across one generation in male mosquitoes	0.0212	[1]
$p^M{}_H$	Probability of wildtype allele converting to H allele across one generation in male mosquitoes	0.979	[1]
$p^F{}_W$	Probability of wildtype allele staying intact across one generation in female mosquitoes	0.0015	[1]
$p^F{}_H$	Probability of wildtype allele converting to H allele across one generation in female mosquitoes	0.985	[1]
$p^{HH}{}_W$	Probability of wildtype allele staying intact across one generation in gravid, homozygous female mosquitoes	0.938	[1]
$p^{HH}{}_R$	Probability of wildtype allele converting to R allele across one generation in gravid, homozygous female mosquitoes	0.0122	[1]
$p^{HH}{}_B$	Probability of wildtype allele converting to B allele across one generation in gravid, homozygous female mosquitoes	0.0437	[1]
$p^H{}_W$	Probability of wildtype allele staying intact across one generation in gravid, hemizygous female mosquitoes	0.997	[1]
$p^H{}_R$	Probability of wildtype allele converting to R allele across one generation in gravid, hemizygous female mosquitoes	0.0007	[1]
$p^H{}_B$	Probability of wildtype allele converting to B allele across one generation in gravid, hemizygous female mosquitoes	0.0017	[1]
b_{WW}	Wildtype mosquito-to-human transmission probability	0.55	[1]
b_H	TP13 drive mosquito-to-human transmission probability	0	[1]
c	Human-to-mosquito transmission probability	0.15	[1]
**Vector biology:**			
β	Egg production per adult female (per day)	21	[2]
T_E	Mean duration of egg stage (days)	3	[2]
T_L	Mean duration of larval stage (days)	7	[2]
T_P	Mean duration of pupal stage (days)	1	[2]

$\mathrm{CV}(T_E)$	Coefficient of variation, egg stage	0.2	[3]
$\mathrm{CV}(T_L)$	Coefficient of variation, larval stage	0.3	[3]
$\mathrm{CV}(T_P)$	Coefficient of variation, pupal stage	0.2	[3]
K	Larval carrying capacity	Time-varying	[4]
μ	Adult mosquito mortality rate	Time-varying	[4]
f	Blood feeding rate	1/3	[5]
Q	Human blood index	0.9	[5]
**Vector control:**			
θ_B	Bites taken on humans while they are in bed as a proportion of all bites taken on humans	0.89	[6,7]
θ_I	Bites taken on humans while they are indoors as a proportion of all bites taken on humans	0.97	[6,7]
r_{LLIN}	Probability of repeating a feeding attempt in the presence of long-lasting insecticide-treated nets	0.56	[6,7]
r_{IRS}	Probability of repeating a feeding attempt in the presence of indoor residual spraying	0.60	[6,7]
s_{LLIN}	Probability of feeding and surviving in the presence of long-lasting insecticide-treated nets	0.03	[6,7]
s_{IRS}	Probability of feeding and surviving in the presence of indoor residual spraying	0	[6,7]
**Intervention setting and demography:**			
N_H	Human population size	223,000	[8]
$PfPr$	All-ages *P. falciparum* prevalence	0.02	[9]
χ_{LLIN}	Proportion of population using long-lasting insecticide-treated nets	0.62	[8]
χ_{IRS}	Proportion of population using indoor residual spraying	0.665	[8]
f_T	Proportion of population using artemisinin-based combination therapy	0.02	[8]

References:

1. Carballar-Lejarazú R, Dong Y, Pham TB, Tushar T, Corder RM, Mondal A, Sánchez C. HM, Lee HF, Marshall JM, Dimopoulos G, James AA (2023) Dual effector population modification gene-drive strains of the African malaria mosquitoes, *Anopheles gambiae* and *Anopheles coluzzii*. Proc Natl Acad Sci USA 120:e2221118120.
2. White MT, Griffin JT, Churcher TS, Ferguson NM, Basáñez MG, Ghani AC (2011) Modelling the impact of vector control interventions on *Anopheles gambiae* population dynamics. Parasites & Vectors 4(1):153.
3. Bayoh MN, Lindsay SW (2003) Effect of temperature on the development of the aquatic stages of *Anopheles gambiae* sensu stricto (Diptera: Culicidae). Bull Entomol Res 93(5):375–81.
4. Winskill P (2022) umbrella: Rainfall & seasonality. R package version 0.2.0.
5. Smith DL, Ellis McKenzie F (2004) Statics and dynamics of malaria infection in *Anopheles mosquitoes*. Malar J 3(1):13.
6. Le Menach A, Takala S, McKenzie FE, Perisse A, Harris A, Flahault A, Smith DL (2007) An elaborated feeding cycle model for reductions in vectorial capacity of night-biting mosquitoes by insecticide-treated nets. Malar J 6(1):10.

7. Griffin JT, Hollingsworth TD, Okell LC, Churcher TS, White M, Hinsley W, Bousema T, Drakely CJ, Ferguson NM, Basáñez MG, Ghani AC (2010) Reducing *Plasmodium falciparum* Malaria Transmission in Africa: A Model-Based Evaluation of Intervention Strategies. PLoS Med 7(8): e1000324.
8. World Bank. São Tomé and Príncipe (https://data.worldbank.org/country/sao-tome-and-principe).
9. World Health Organization Global Health Observatory (https://www.who.int/data/gho).
